# Thermal Development, Mortality, and Fertility of an Apulian Strain of *Drosophila suzukii* at Different Temperatures

**DOI:** 10.3390/insects16010060

**Published:** 2025-01-10

**Authors:** Nuray Baser, Luca Rossini, Gianfranco Anfora, Kürşat Mustafa Temel, Stefania Gualano, Emanuele Garone, Franco Santoro

**Affiliations:** 1 International Centre for Advanced Mediterranean Agronomic Studies of Bari (CIHEAM Bari), 70010 Valenzano, Italy; baser@iamb.it (N.B.); kursat.temel51@gmail.com (K.M.T.); gualano@iamb.it (S.G.); 2 Service d’Automatique et d’Analyse des Systèmes, Université Libre de Bruxelles, 1050 Brussels, Belgium; luca.rossini@ulb.be (L.R.); emanuele.garone@ulb.be (E.G.); 3 Center for Agriculture, Food and Environment (C3A), University of Trento, San Michele all’Adige, 38098 Trento, Italy; gianfranco.anfora@unitn.it

**Keywords:** insect rearing, life history, pest modelling, population dynamics, spotted wing drosophila, thermal development

## Abstract

This study explored the thermal response of *Drosophila suzukii*, an injurious insect pest present in many countries worldwide, at different controlled conditions. This species is responsible for several economic losses in soft fruit cultivations, develops on ripening fruits, and has the capability to quickly adapt to new territories and climates, closing multiple generations per year. Given its high invasive potential and the increasing need for low-impact control strategies, an in-depth exploration of the biology of this species and of the stage thermal response is fundamental. Specimens of an Italian strain from Apulia were reared in growth chambers at different constant temperatures (6, 9, 13, 18, 20, 24, 25, 26, 27, 28, 29, 31, 32 and 33 °C). The life cycle of each specimen was individually tracked from the egg to the death of the adults, considering the larval stages distinction as well. Besides development and mortality, egg production over temperature has been recorded. The dataset was first analysed according to life tables studies; then, we also estimated the biological parameters of the most common equations describing development, mortality, and fertility involved in physiologically-based model applications. The results confirmed and extended the information on the thermal response already present in the literature, but with reference to a population adapted to warmer climates. The species successfully developed from egg to adult at 13–29 °C, while between 6–9 and 29–33 °C the development was limited to L2/L3 stages. Optimal temperatures are around 26–28 °C, depending on the life stage. This study provides one of the complete overviews of the thermal response of *D. suzukii*, which is available in the current literature, and opens the door to more accurate modelling frameworks.

## Simple Summary

Exploring insects’ biology through growth chamber experiments is a common practice that provides quantitative information on the stage-response of the species to external factors. Those experiments are commonly indicated as “Life tables studies” and provide for rearing a cohort of eggs under different conditions of temperature, relative humidity, diet, and photoperiod, to cite some examples. Tracking the life history of the individuals, it is possible to assess how the growth conditions affect the stage-development time, the stage-mortality, and the reproduction rate (egg production, pre-oviposition period, and coupling). Besides the biological knowledge, the quantitative information resulting from life table studies allows the parameter estimation of physiologically-based models. For this reason, life table studies are widely applied to insect pests, and they represent a piece of fundamental knowledge that can potentially drive integrated management programmes.

## 1. Introduction

The spotted wing drosophila, *Drosophila suzukii* (Matsumura) (Diptera: Drosophilidae), is an insect pest infesting soft fruit cultivations, causing several economic damages in the food industry. Many soft fruit orchards (e.g., cherry, blueberry, strawberry, and peach) are experiencing several economic losses that are both due to the reduction of the yield and the increase of management costs [1,2,3]. Its high adaptability to different environments and different host plants contributed to its spread from Southeast Asia (Japan), its place of origin, to different countries in Western Asia, Europe, North and South America, and, recently, in Africa [2,3,4,5]. In favourable conditions, this pest can close up to 15 generations, an aspect that makes the infestations in cultivated fields extremely hard to contain [6,7]. For this reason, several studies have been conducted over the years on the biology and ecology of this species, on monitoring techniques, and on alternative control methods (i.e., natural enemies or biopesticides) [7,8,9,10,11,12,13,14,15,16,17,18,19,20].

The life cycle of *D. suzukii* is composed of an egg stage, three larval instars, a pupal stage, and a female and male adult. Females are more difficult to recognise without using a stereomicroscope since they are closely similar to *D. melanogaster* (Meigen) (Diptera: Drosophilidae) except for the shape of the serrated and sclerotised ovipositor [2]. Conversely, adult males are characterised by two black spots on the wings that can be seen with the naked eye and determine the common name of the species as spotted wing drosophila (SWD) [2]. The characteristic spots favour a first fast identification during field monitoring surveys [6]. It should be noted, however, that several other *Drosophila* spp. have spots on the wings, such as *D. biarmipes*, *D. rajasekari* and *D. subpulchrella* [21,22,23,24]. The male of *D. suzukii* is also characterised by having two combs on the tarsal [2,8].

The damage is caused by different life stages. Females can easily penetrate the fruit skin through their serrated ovipositor and lay eggs [25], leading to infections by secondary pathogenic agents (e.g., fungi, yeast and spoilage bacteria) or modifying the shape of the fruit, with a subsequent decrease of the market value [26,27,28]. Larvae, instead, feed and develop into fruits, leading to a direct reduction of the yield [1,3]. The harmfulness of this pest, either in more temperate areas or in climate change scenarios, is increased by the overwintering phase as well [29]. *Drosophila suzukii*, in warmer areas, overwinters as an adult, with a subsequent higher proportion of specimens that start the first generation of the following season [30].

As with any arthropod, the duration of the life cycle, as well as part of mortality and egg production, depends on the environmental conditions, such as temperature [31,32]. Over the years, mathematical models for forecasting the life cycle of insects were arranged based on life cycle data influenced by environmental factors called “life tables”. The life tables provide data on the life history of each specimen until its death in the adult stages [15,33,34,35,36,37,38,39,40,41,42,43,44].

The first life tables study for *D. suzukii* populations was carried out by Tochen et al., [44], who explored the development of this species under different constant temperatures by focusing on cherry and blueberry as host plants. Temperature-dependent mortality and fertility, instead, were investigated by Kinjo et al., [45] and Ryan et al., [46], and those papers constitute the basis for more quantitative knowledge on *D. suzukii* thermal development. This piece of information was widely used for the parameterisation of physiologically-based models [35,37,38,39,47] that have been proposed as kernels of decision support system tools.

Given the recent restrictions in terms of pesticide uses that many countries worldwide are adopting [48,49,50,51], decision support systems are gaining importance for the optimisation of resources [51,52]. Field monitoring, in fact, is not sufficient anymore to establish where and when treatments should be carried out, above all in the modern framework of precision agriculture [38]. Proper planning of the control strategies can be supported by mathematical models that reproduce in silico the future scenarios of the field infestations and, in the more accurate systems, the effect that a given treatment can potentially have [53].

The high adaptability of *D. suzukii* to different environments may provoke a modification of life table parameters and of the physiologically-based models from them, leading to a reduction of the predictive potential. Two criticalities can be identified in the current literature on *D. suzukii*: (*i*) the lack of an in-depth life tables study for European (or Mediterranean) populations, and (*ii*) up-to-date life tables have divided the *D. suzukii* life cycle into egg, larva (without distinction between the larval instars), and pupa, and they do not account for the adult survival.

This study aims to extend the quantitative information on the biology of this pest, as well as to increase the resolution of life cycle description, through a complete study of life tables that analyse the response to temperature changes of development, survival, and fecundity of a European population of *D. suzukii*. We focused on a population collected in the Apulia region (South Italy): from continuous rearing, we obtained a cohort of eggs that were placed in climatic chambers at different temperature conditions (6, 9, 13, 18, 20, 24, 25, 26, 27, 28, 29, 31, 32, and 33 °C), and individually followed until the death of the adults. A second part of the study, instead, explored egg production over the same temperature conditions. Besides presenting results according to the classical representation of life tables studies, we also estimated the biological parameters of the most common equations describing development, mortality, and fertility involved in physiologically-based model applications.

## 2. Materials and Methods

### 2.1. Drosophila suzukii Wild Specimens’ Origin and Continuous Rearing System

Apulian wild populations of *D. suzukii* were collected on 11 October 2012 from the organic vineyard of CIHEAM Bari (41.053674, 16.877897 E) [54]. Continuous rearing of individuals was started within the insectarium facilities of CIHEAM Bari, set at T 23 ± 1 °C, 65% RH, and 16:8 L:D. We periodically added fresh adults collected from orchards (wild type) to the *D. suzukii* rearing chamber to avoid problems of outbreeding [13]. After identification, adult specimens were subsequently moved to ventilated Plexiglas cages (50 × 40 × 40 cm) containing a Petri dish with a diet medium substrate (Section 2.2). The plates served as a food source (only for adults) and as an oviposition area, but to extend the oviposition surface, blueberries were also provided as a host crop. Wetted tissues provided a water source.

### 2.2. Diet Medium Preparation and Growth Chambers

**Figure 1:**
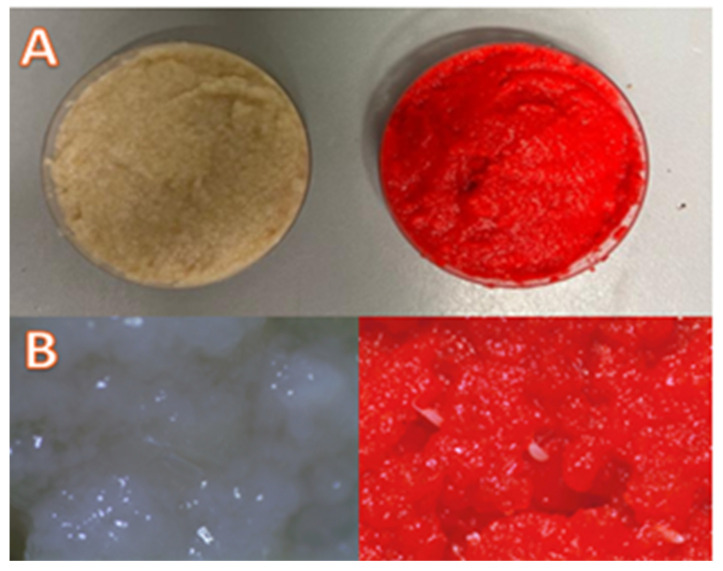
Diet media for *Drosophila suzukii* rearing. (**A**) General appearance of the diet media as non-coloured and coloured. (**B**) Diet media with eggs under the microscope (with and without colourant, respectively).

The diet medium was the same either for the continuous rearing or for the rearing at the different constant temperatures, and it was prepared as follows: 58.8 g of corn flour, 58.8 g of white sugar, 76.5 g yeast, 2.06 g of methyl-4-hydroxybenzoate and 3.5 g of agar meshed in 1 litre of boiling distilled water. The boiling process lasted for 30 min, during which the mixture was stirred every two minutes. Subsequently, the mixture was cooled to 25 °C, after which 1.76 mL of 99% propionic acid was added (N. Baser formulation, unpublished). The mixture was stored at 4 °C in vials. Before use, the substrate was deployed on Petri dishes and coloured with a red colourant for food that allowed easier differentiation of *D. suzukii* eggs (Figure 1).

Three growth chambers (FDM-Environment Makers, Rome, Italy), located in the laboratory of the Innovative Approaches for IPM of Mediterranean Fruit and Vegetable Crops department of CIHEAM Bari, were equipped with neon lighting programmed for a 16 h light and 8 h dark cycle (L:D = 16:8) and a data logger (EL-USB-2+, Lascar Electronics, Whiteparish, UK) to double-check the climatic conditions. The photoperiod was set according to the experimental conditions described by Tochen et al. [44], and the relative humidity (RH) was maintained at a constant value of 70 ± 5%. Temperatures, instead, were set at 6, 9, 13, 18, 20, 24, 25, 26, 27, 28, 29, 31, 32, 33 (±1) °C.

### 2.3. Egg to Adult Development

For each experiment, newly laid eggs were collected by continuous rearing, as described in Section 2.1. A fresh Petri dish containing a diet medium substrate was introduced into each of the rearing cages containing adults for 3 h. Subsequently, 50 eggs were picked and individually transferred to new Petri dishes containing red-coloured substrate and moved to the growth chamber set at the specific temperature for the experiment.

The Petri dishes (containing one egg/dish), properly labelled, were observed at regular time ranges that depended on the temperature of rearing: at 6, 9, and 13 °C, inspections were carried out every 24 h; at 18 and 20 °C, inspections were carried out every 8 h; at 24, 25, 26, and 27 °C, every 6 h; at 28 and 29 °C, every 4 h; and at 31, 32, and 33 °C, every 2 h. Temperature-dependent sampling times were set based on experience and empirical estimations carried out during data collection. During the inspection, the life stage (egg, L1, L2, L3, pupa, adult males, and adult females) and the status (dead/alive) of each individual was noted using the datasheet template and the guidelines available at https://github.com/lucaros1190/LifeTables-StandardSpreadsheet (accessed on 3 January 2025). Each experiment ended with the death of the final out of the 50 individuals of the cohort: at some temperatures, none of the specimens reached the adult stage, while where it was reached, we tracked the adult longevity as well. The larval stage identification was based on morphological traits, following the guidelines and the dichotomous keys in [55].

### 2.4. Egg Production over Temperature

Where temperatures allowed development up to the adult stage, we selected 10 couples of newly emerged adults. Each couple was subsequently transferred to an oviposition assessment jar containing a diet medium substrate for food, a piece of wetted tissue for water, and a host fruit (blueberry) for oviposition. The substrate and host fruits were replaced once per day, and the number of newly laid eggs by each couple was recorded. Additionally, the water source was renewed daily to prevent potential infections. The remaining adults, apart from the 10 couples kept in the jar to assess egg production, were reared individually in plastic cups with food and a water source and served as a reserve of virgin specimens in case of male mortality in the replication jars. If males died, a new virgin male was introduced into the jar containing the female, and the study continued until the female’s death. It is worth noting that the couples involved in the egg production trial developed under the same temperature as the preimaginal stages. In other words, each constant-temperature experiment covered the whole generation time, from egg to egg production from adults.

### 2.5. Life Tables Analysis

#### 2.5.1. Development and Mortality

The development times of the individuals in the different life stages and at different temperatures were further analysed to build the life tables. In this phase, we followed the analysis of [43]: for each life stage and constant temperature, we calculated the mean, standard deviation, median, mode, skewness, kurtosis and synthetic indicators that provide a complete overview of the shape of the distribution of the values. This part of the analysis also included adult males and females. Temperature-dependent mortality, instead, was expressed as the percentage of relative individuals that did not pass to the next stage. This part of the analysis did not include adults, as their longevity had been considered in the previous step.

Before moving ahead to the next section, it is worth pointing out that, for the sake of this study, we focused on the differential representation of the life tables as being more suitable for our goals. The cohort/integral representation, as the one described by [39,40], has, however, been reported as Supplementary Materials (in the raw dataset) and at https://github.com/lucaros1190/DSuzukiiLifeTables (accessed on 3 January 2025), as it may be helpful to the scientific community for further research. The dataset, in fact, has been collected using a spreadsheet that aims to become a standard for life table experiments, endorsing data collection and sharing.

#### 2.5.2. Pre-Oviposition Period and Egg Production over Temperature

For each constant temperature, we calculated the average time and the corresponding standard error, expressed in days, from female emergence to the first oviposition. The average includes the overall values assessed for the 10 females reared separately, as described in Section 2.4; the data were realigned by considering the day of coupling as time zero.

The average total number of eggs produced by females at different temperatures was calculated as the average of the total eggs produced by each specimen with the standard error. In the Supplementary Materials, we report the distribution of eggs produced per female per day. The distribution was obtained by calculating the average number of eggs produced by the 10 females for each day.

### 2.6. Temperature-Dependent Development, Mortality, and Fertility Rate Functions

Life table data can also help estimate the parameters of mathematical functions that relate development, mortality, and fertility to the temperature of the living environment. Given the importance of these functions in the formulation of physiologically-based models [36], we provided the best-fit parameters for the most common ones.

#### 2.6.1. Temperature-Dependent Development Rates—Preimaginal Stages

The stage-development times $D_{i}(T)$ of each individual at different constant temperatures can be converted into rates $G_{i}\left(T\right)$ according to the following expression [56,57,58]:

(1) $$G_{i}\left(T\right)=\frac{1}{D_{i}\left(T\right)}$$

The expression (1) transforms the data from a decreasing-increasing to an increasing-decreasing profile, defining a maximum that theoretically coincides with the optimal temperature for the development and a lower and upper threshold below and above which the development is theoretically not allowed, respectively.

The reshaped dataset can be described by manifold mathematical functions that have been proposed over the years by different authors. Since a great portion of the equations available have been formulated on an empirical basis, for the sake of this study, we considered only the most common ones, plus the Sharpe and De Michele equation, which includes thermodynamic parameters. Proceeding in order, we considered the following:

- The Logan development rate function [59]

(2) $$G(T)=\psi \left[exp\left(\rho T\right)-exp\left(\rho T_{M}-\frac{T_{M}-T}{\Delta T}\right)\right]$$

where ψ and ρ are empirical parameters, $T_{M}$ is the maximum temperature above which development is theoretically not possible, and ΔT is the temperature range between the maximum of the function $G\left(T\right)$ and $T_{M}$.

- The Briére development rate function [60]:

(3) $$G(T)=aT\left(T-T_{L}\right){\left(T_{M}-T\right)}^{\frac{1}{m}}$$

where a and m are empirical parameters, and $T_{L}$ and $T_{M}$ are the lower and maximum temperature thresholds below and above which development is theoretically not possible, respectively.

- The Sharpe and De Michele rate function [61,62]:

(4) $$G(T)=\frac{T\exp\left(A-\frac{B}{T}\right)}{1+\exp\left(C-\frac{D}{T}\right)+\exp\left(E-\frac{F}{T}\right)}$$

where A, B, C, D, E, and F are parameters related to the enzyme kinetics [48].

- The Lactin-1 rate function [63]:

(5) $$G\left(T\right)=\exp\left(a\;T\right)-\exp\left[a\;T_{M}-\left(\frac{T_{M}-T}{∆T}\right)\right]$$

where $T_{M}$ is the maximum temperature above which development is theoretically not possible, ΔT is the temperature range between the maximum of the function $G\left(T\right)$ and $T_{M}$, and *a* is an empirical parameter.

- The Lactin-2 rate function [63]:

(6) $$G(T)=\exp\left(a\;T\right)-\exp\left[a\;T_{M}-\left(\frac{T_{M}-T}{∆T}\right)\right]+\lambda$$

where $T_{M}$ is the maximum temperature above which development is theoretically not possible, and ΔT is the temperature range between the maximum of the function $G\left(T\right)$ and $T_{M}$, and *a* and λ are empirical parameters.

#### 2.6.2. Temperature-Dependent Mortality Rates—Preimaginal Stages

The survival rates of each stage at the different constant temperatures, $S_{i}(T)$ can be converted into mortality rates, considering that $M_{i}(T)=1-S_{i}(T)$. The dataset, organised in this way, provides a typical profile with values close to one as the temperature approaches the thermal limits (lower and upper) and close to zero around the optimal temperature for development. As for the development rates, over the years, different authors have proposed some empirical functions that interpolate this peculiar profile. We followed the same approach discussed in Section 2.6.1, by estimating the parameters of the most common functions offered by the current literature. The equations in this case are as follows:

- The “bathtub function” of Wang et al. [64]:

(7) $$M_{i}(T)=a_{m}T^{4}+b_{m}T^{3}+c_{m}T^{2}+d_{m}T+e_{m},$$

a fourth-order polynomial equation where $a_{m}$, $b_{m}$, $c_{m}$, $d_{m}$, and $e_{m}$ are empirical parameters with no direct biological meaning.

The mortality rate of Kim and Lee [65], subsequently revised by Son and Lewis [66]:

(8) $$M_{i}(T)=1-\left[k\exp\left(1+\frac{T_{MAX}-T}{\rho_{T}}-\exp\left(\frac{T_{MAX}-T}{\rho_{T}}\right)\right)\right]$$

where k and $\rho_{T}$ are empirical parameters, and $T_{MAX}$ is the temperature at which mortality is lower. In line of principle, $T_{MAX}$ may coincide with the optimal temperature for the development, but there are cases of species where those values can be shifted [67].

#### 2.6.3. Temperature-Dependent Adult Longevity

Adult male and female longevity over temperature, which can also be referred to as survival, has been fitted using either the equations listed in Section 2.6.1, (2)–(6), or the bathtub function (7). Preliminary attempts were carried out to test the (8) as well, but for the sake of this part of the study, it has not been considered due to the lack of convergence of the fitting algorithms (Section 2.6.5).

#### 2.6.4. Temperature-Dependent Fertility Rates

Fertility, in this study, is defined as the total number of eggs produced per female over temperature, as already explained in Section 2.4. Temperature-dependent fertility has an increasing-decreasing profile, with a maximum coinciding with the optimal temperature for egg production. For the other rates, even in this case, only empirical functions interpolate the dataset, and the choice is extremely reduced. We hereby use the Gaussian-like equation introduced by [46]:

(9) $$\beta (T)=\alpha \left[\frac{\gamma +1}{\pi \lambda^{2\gamma +2}}{\left(\lambda^{2}-\left({\left[T-\tau \right]}^{2}+\delta^{2}\right)\right)}^{\gamma }\right]$$

where α, γ, λ, and δ are empirical parameters, and τ is the optimal temperature for egg production. This equation has already been applied to a North American population of *D. suzukii*, allowing a direct comparison of the results.

#### 2.6.5. Parameters Estimation and Software

The best-fit parameters were estimated through non-linear least-squares regression carried out using MATLAB (v. 2023b) software. An evaluation of the fitting performances of the functions (2)–(6) was carried out by considering the coefficient of determination $R^{2}$, the root mean square error (RMSE), and through a $\chi^{2}$-test as detailed in [57,68]. The script and the raw dataset to fully reproduce the results of this study are available at https://github.com/lucaros1190/DSuzukiiLifeTables (accessed on 3 January 2025).

## 3. Results

### 3.1. Life Tables: Development and Survival over Temperature

The life table values corresponding to the stage development and survival at different constant temperatures are reported in Table 1. Proceeding by order, the only stage that could develop, even with a few individuals, under all the temperature conditions tested, was the egg. The longest and shortest development times for the egg stage were recorded at 6 °C (9 ± 1 days) and 26–27 °C (0.8 ± 0.2–0.8 ± 0.3 days), respectively. As expected, the mortality rate was high (74%) at the lowest tested temperature and low (0.96 and 0.88%) at the optimal temperature. At 33 °C, instead, egg mortality was extremely high (94%), even if the development time was still short compared with the other temperatures (1.75 ± 0.08 days).

First instar larvae (L1) showed a trend similar to that of eggs, but no individuals survived up to L2 at 33 °C, which is considered the temperature with the highest relative mortality. The development times ranged from 6.3 ± 0.6 days at 6 °C to 0.8 ± 0.2 days at 28 °C, while the lowest relative mortality rate was observed at 26 °C (5%). Second instar larvae (L2) successfully reached the L3 stage in the temperature range of 9–31 °C, showing longer and shorter development times at 9 °C (5.8 ± 0.4 days) and 28 °C (1.1 ± 0.2 days), respectively. A lower relative mortality rate in this case was observed at 26 °C, where only 5% of the individuals died. Third instar larvae (L3) successfully developed up to pupa in the temperature range of 9–31 °C, as in the case of L2, with shorter and longer development times at 9 °C (5.8 ± 0.4 days) and 27–28 °C (1.4 ± 0.2–1.4 ± 0.3 days), respectively. We observed zero relative mortality at 20, 25, and 26 °C, while the highest percentage was at 31 °C (57%). Pupa showed the longest development time for all temperatures among the preimaginal stages. No relative mortalities were assessed, except at 29 °C and 31 °C. The individuals successfully developed up to the adult stage in the range 13–29 °C, with shorter and longer development times at 13 (13.1 ± 0.9 days) and 26 °C (4.4 ± 0.3 days).

Overall, the values in Table 1 show that temperatures close to the thermal limits affect individuals more as they advance in life stages. In other words, the egg stage seems to be much more resistant to thermal stresses with respect to the adults and the preimaginal stages, while the stage with the lowest relative mortality was the pupa, with values close to 1 for all temperatures.

### 3.2. Life Tables: Adult Longevity

Adult longevity was described in the same way as preimaginal stages, except for the survival rate, which, in this case, has no sense. Both males and females developed in the temperature range of 23–29 °C (Table 2), with differences among the development times more reduced with respect to the preimaginal stages. Adult males showed exceptional longevity at 20 °C, with an average value of 40 ± 10 days. Conversely, this value was not observed at the same temperature for females, who reported values that were in line with the overall dataset.

It is worth pointing out that the standard deviations of the times were higher than in the preimaginal stages, highlighting a higher inhomogeneity between the specimens reared. Additionally, the longevity of males was longer than that of females in the overall different constant temperatures.

### 3.3. Pre-Oviposition and Egg Production over Temperature

Egg production was observed in the range of 13–29 °C, namely, all the temperatures discussed in Section 3.2, where females could develop. The pre-oviposition period ranged from 1 ± 1 to 2.8 ± 0.4 days, with limits observed at 24 and 29 °C, respectively (Table 3). In terms of eggs produced, the highest average total number was observed at 24 °C, where the pre-oviposition period was also the lowest recorded, while the lowest egg laying was observed at 13 °C. Overall, egg production was lower at low temperatures, even if the longevity of the adult females (Section 3.2) was comparable.

### 3.4. Temperature-Dependent Stage-Development Rate Functions of the Preimaginal Stages

**Figure 2:**
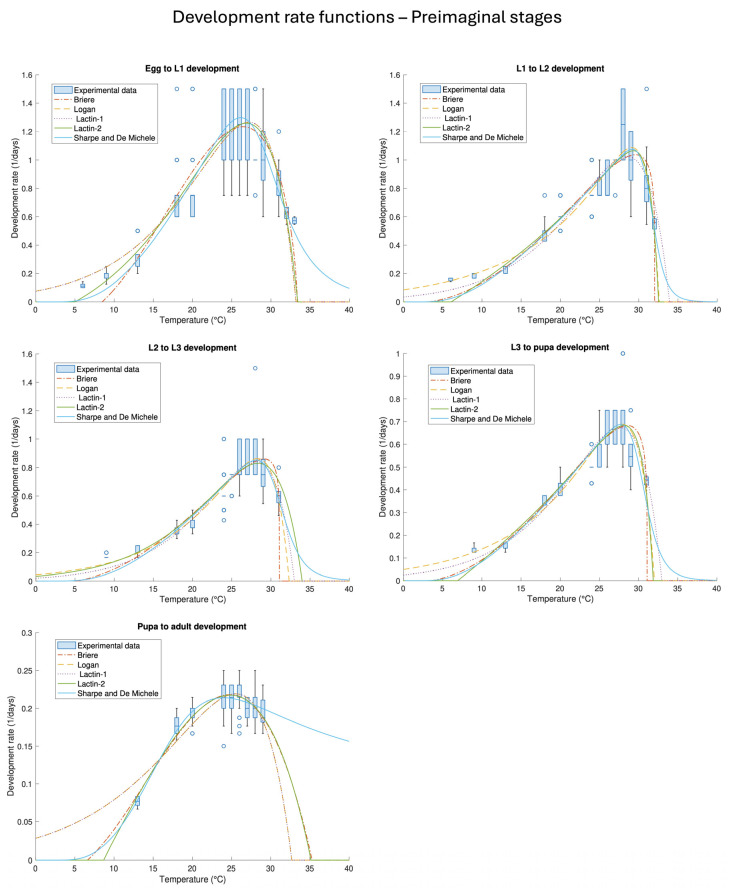
Best-fit temperature-dependent development rate functions (2)–(6) and experimental data of the preimaginal life stages (egg, L1, L2, L3, and pupa). The best-fit parameters and their corresponding standard errors are listed in Table A1.

The second part of the results concerns the mathematical interpretation of life table data. The preimaginal stages (egg-pupa) were overall well represented by the development rate functions (2)–(6), as graphically shown in Figure 2. The parameters and standard errors (SE) of the best-fitting functions are listed in Table 4, and a complete list of the results is reported in Table A1. Proceeding in order, the dataset of the egg stage was best represented by the Sharpe and De Michele (4) and Lactin-2 (6) equations. The dataset was overall scattered compared to the other life stages, in particular, around the optimal temperatures (boxplot in Figure 2); as a consequence, the $\chi^{2}$ value was lower. The optimal temperatures for the development of the species, namely the abscissae corresponding to the maxima of the equations, are centred around 26 °C (Sharpe and De Michele) and 27 °C (Lactin-1). The minimum temperature below which egg development is theoretically not possible is indicated by the Brière Equation (3) as 8 ± 1 °C. Conversely, the upper thermal threshold is a parameter included in manifold equations (Logan, Brière, Lactin-1, and Lactin-2), and the values are in accordance with each other: they range from 33.2 ± 0.3 (Logan) to 33.9 ± 0.6 (Lactin-2).

A slightly different scenario was observed for the L1 stage. The best-fit performance was obtained using the Logan equation (Table 4), even if the difference with respect to the other equations was minimal, analogous to eggs. The dispersion of the dataset (boxplot in Figure 2) was lower than that of the eggs and led to an overall higher coefficient of determination $R^{2}$. The optimal temperature for development is slightly higher than that of eggs and ranges between 28 and 29 °C, with the lowest and highest values calculated for Lactin-1 and Brière, respectively. The minimum thermal threshold, according to Equation (3), was 3 ± 2 °C, while the maximum ones ranged from 32.02 ± 0.03 °C (Brière) to 33.9 ± 0.3 °C (Lactin-1).

The development of L2 was best represented by the Logan equation (Table 4), even if the difference in terms of goodness of fit was not much higher than that of the other equations. Dataset dispersion was reduced compared to the other stages (boxplot in Figure 2), and the optimal temperatures ranged between 28 °C (Lactin-1) and 29 °C (Brière), analogous to the L1 stage. The minimum thermal threshold is 6 ± 1 °C, while the maximum range is between 31.07 ± 0.04 °C (Brière) and 34.0 ± 0.6 °C (Lactin-2).

A similar scenario was observed for L3, where the dispersion of the dataset was not high (boxplot in Figure 2), and the best-fit performance was obtained using the Logan as well as Sharpe and De Michele equations (Table 4). The goodness of fit parameters were slightly above the other functions, in line with the situation observed for the previous preimaginal stages. The optimal temperatures range between 27 °C (Lactin-1) and 28.5 °C (Brière), while the lower thermal threshold indicated by the Brière equation was 3 ± 2 °C, as for L2. The upper thermal thresholds range from 31.08 ± 0.09 °C, observed for the Brière equation, to 33.0 ± 0.2 °C, observed for the Lactin-1. Pupa showed a different trend than the previous stages (Figure 2): we observed higher development rates even towards the thermal limits, with an overall dataset that was not highly dispersed. The best-fit performance was obtained by Sharpe and De Michele (4), closely followed by others (Table 4). However, Equation (4) seems to predict unreliable development rate values as temperature increases, leading us to discard this result in favour of the Lactin-2 equation, second in terms of goodness of fit. The optimal temperatures for the development of pupae ranged from 24 (Brière and Lactin-2) to 25.5 °C (Lactin-1), while the lower thermal threshold was 7 ± 1 °C, according to the Brière equation [60]. The upper thermal threshold, instead, ranged from 35 ± 2 °C (Brière) to 45 ± 8 °C (Lactin-2) [63].

It is worth pointing out that the Sharpe and De Michele Equation (4) showed much higher upper thermal thresholds than the other functions; those values look unreliable for constant-temperature development, as also suggested by the experimental data. The second issue related to fitting Equation (4) was the estimation of the standard errors of the parameters; values were totally unreliable and, for this reason, were not reported. Correlations between parameters and difficult fit performances of Sharpe and De Michele are well known [61], but since, to date, it is the only non-empirical equation that describes temperature-dependent development, it is worthy of exploration.

### 3.5. Temperature-Dependent Stage-Mortality Rate Functions of the Preimaginal Stages and Adult Survival/Longevity

**Figure 3:**
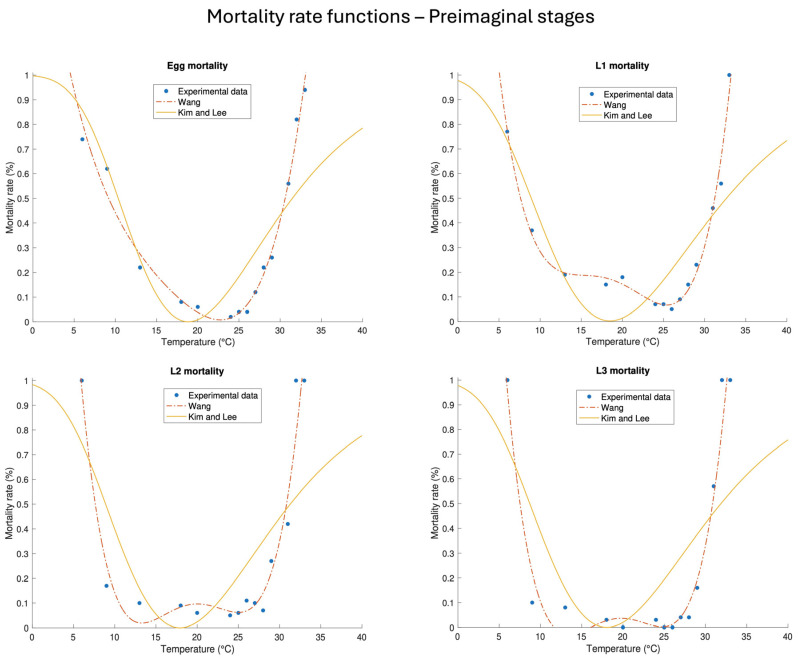
Best-fit temperature-dependent mortality rate functions (7)–(8) and experimental data of the preimaginal life stages (egg, L1, L2 and L3). The best-fit parameters and their corresponding standard errors are listed in Table 5.

The second step of the mathematical interpretation of the life tables concerned the temperature-dependent mortality rates of the preimaginal stages. The parameters of Equations (7) and (8) were estimated for egg, L1, L2, and L3, but not for pupa, given that the values listed in Table 1 did not show any suitable trend for this stage. The bathtub Equation (7) had a better fit performance than Equation (8) for all preimaginal stages, as highlighted by the goodness of fit values listed in Table 5. The difference can also be visually observed in the plots in Figure 3, and a complete list of the results is reported in Table A2.

Proceeding by order, Equation (7) indicated the lowest temperature-dependent mortality rate for eggs at around 22.5 °C, while Equation (8) was at 19 ± 1 °C. The difference was more evident in L1: the lowest value of the best-fit Equation (7) was around 26 °C, while that of Equation (8) was 18 ± 1 °C. The trend of the best-fit Equation (7) for stages L2 and L3 was different from that for eggs and L1, as there were two minima instead of one around 12 and 25 °C for L2 and 13 and 25 °C for L3. The latter showed unreliable mortality rate values (negative) in the range of 11.5–16.3 °C, leading us to discard this function in favour of Equation (8) for this stage. Conversely, the lower mortality rates indicated by Equation (8) for L2 and L3 were 18 ± 1 and 18 ± 2 °C, respectively.

**Figure 4:**
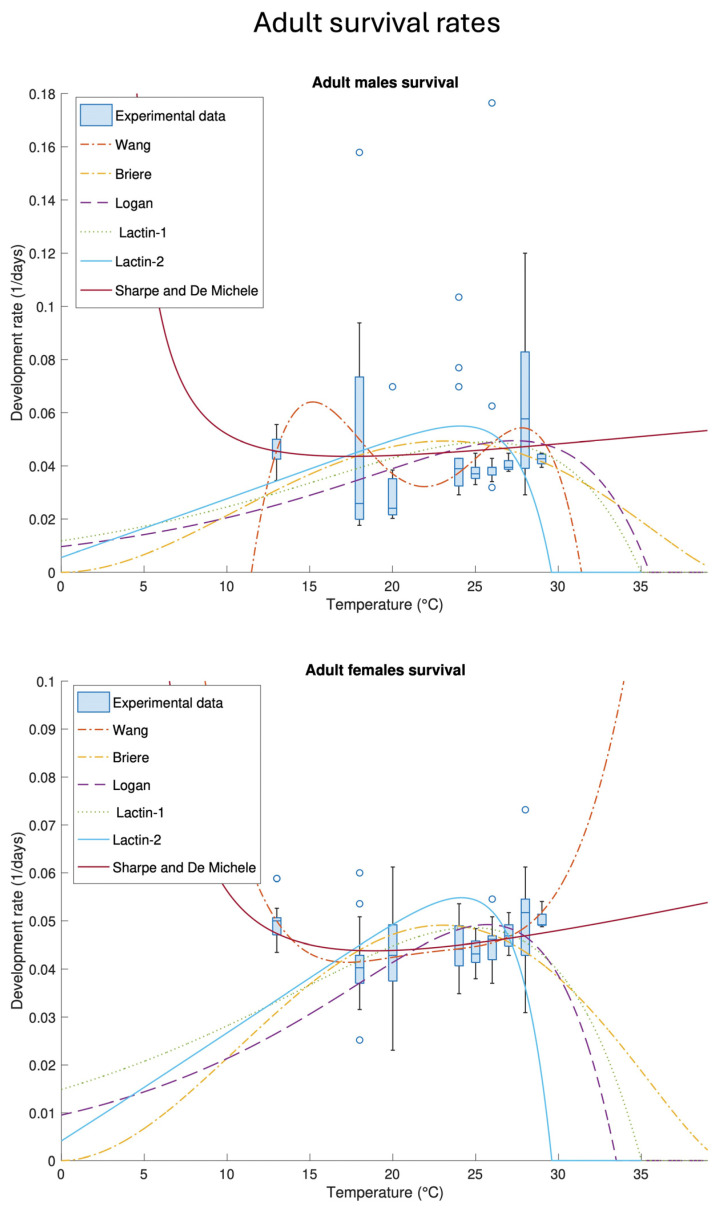
Best-fit temperature-dependent survival rate functions (2)–(7) and the experimental data of adult males and females. The best-fit parameters and their corresponding standard errors are listed in Table 6.

The best-fit parameters of temperature-dependent adult male and female survival/longevity rates (2)–(7) are listed in Table 6 and plotted in Figure 4. The overall fit performance of the functions involved in this part of the study was low, probably because of the lack of a well-identified trend in the data. In particular, the fit performance was strongly reduced by the experimental dataset at 13 °C, which is far from the best-fit functions. Given the dispersion of the dataset, moreover, we did not consider the $R^{2}$ among the goodness of fit parameters since the values provided were unreliable. Either for males or females, the Sharpe and De Michele Equation (4) provided unrealistic results, while the other functions showed a similar fit performance (Table A3). A slightly higher precision, overall, has been reported by the Logan (2) and Lactin-1 (5) equations for males and only by Lactin-1 for females.

### 3.6. Temperature-Dependent Fertility Rate Function

**Figure 5:**
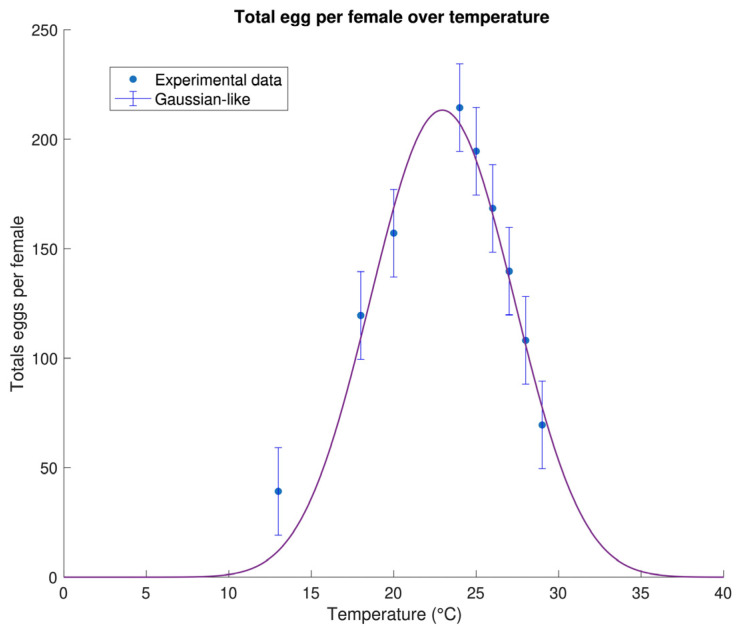
Best-fit egg production rate function (9) and experimental data. The best-fit parameters and their corresponding standard errors are listed in Table 7.

The dataset organised as described in Section 2.5.2, was well described by the Gaussian-like Equation (9). The best-fit parameters and their uncertainties are listed in Table 7, and a graphical representation of this result is provided in Figure 5. Egg production is at a maximum of around 24 °C, with an estimated total amount of approximately 230 eggs/female.

## 4. Discussion and Conclusions

This study analysed the thermal response of an Italian population of *Drosophila suzukii,* providing either a complete life table study or its mathematical interpretation using the most common development, mortality, and fertility rates. To the best of our knowledge, the set of temperatures explored in this study is the most complete compared with the existing literature on this species. The overall dataset also reaches a high level of detail because of the high resolution in the stage-population analysis at different temperatures, which accounts for an optimised sampling time and a high-level distinction of the larval instars. Additionally, the raw dataset is publicly available; to date, it is the only life table dataset for *D. suzukii* in the public domain. Besides the possibility of integrating this dataset with further research, it is also worth pointing out that we focused, to analyse the data, on the differential representation of life tables since the cohort/integral representation was not suitable to pursue the aim of this study.

However, the dataset included in the Supplementary Materials reports this second representation, and it has also been a bench test for the life table standard spreadsheet described in Section 2.3. The spotted wing drosophila deserves attention, as it is one of the most dangerous pests for soft fruit cultivation worldwide. This pest is responsible for the serious reduction in yields, and its high damaging potential is endorsed by its adaptability and capability to survive by exploiting different host species [69]. For this reason, life table studies are extremely important and should be repeated in different populations worldwide to better understand these differences. To date, the most detailed studies that we can use for comparison come from North America in Oregon, more specifically [70]. This area is climatically different from Southeast Italy, as the latter is warmer than North-East America, and is a good example to discuss to understand the adaptability level of this pest.

For instance, it is known that *D. suzukii* does not tolerate high temperatures [71,72] above 29–30 °C; in fact, it is possible to observe either a high mortality of the adults or a reproduction standby. Our data showed that in the Apulian population, taken into account, there was likely a sort of adaptation to high temperatures, as our individuals at 29 °C were still able to develop from egg to adult and to produce a conspicuous number of eggs per female. Toleration to high temperatures has also been recently explored by Horváth et al. [73], and our outcomes are in line with the cited literature. Conversely, low temperatures (6–13 °C) were tolerated by only a few preimaginal stages, as none of the individuals successfully developed over L2–L3 under these conditions. The mathematical interpretation of the life table data provided more precise information on this aspect; L1 and L3 seemed to be more tolerant to low temperatures, while pupae were more tolerant to high temperatures.

The development times of the preimaginal stages reported by Tochen et al. [44] are in line with our results. More specifically, we could directly compare three temperatures (18, 26, and 28 °C), but the others (10, 14, 22 and 30 °C) were close to the experimental conditions we have explored. Unlike Tochen et al., [44], however, we did not observe a gap in development between males and females at high or low temperatures. A possible explanation could be the different diets used; we provided an artificial diet, while in the above-mentioned study, fresh cherries and blueberries were used. It is also worth noting that we carried out sex identification during the adult emergence phase, not before; therefore, we cannot say if there were differences in mortality between males and females in the preimaginal stages.

From the development and mortality rate functions, instead, we noticed a shift between the peaks of almost all the preimaginal stages. The optimal temperature for development, where the life cycle is supposed to be the shortest, does not coincide with the optimal temperature for survival, which was instead 2–3 °C lower. Even if this information comes from a mathematical interpretation of the dataset, it highlights an additional aspect that can contribute to the adaptability of this species. We may conclude, in fact, that the range of 24–28 °C is the optimal one for development, either for low mortality or for fast development (and egg production as well). This fact explains why a higher presence of this pest is observed during late spring and early autumn in areas featuring a Mediterranean climate [5,15,27,54].

Adult longevity also contributes to the maintenance of a large population. While the trend of the development rates over temperature is marked in the preimaginal stages, with low values towards the thermal limits and high values around the optimum, the same trend has not been observed for adult longevity. Even at the lowest temperature explored for adult emergence (13 °C), the survival rate was still high. If this information is combined with outcomes from other researchers on tolerance to extreme temperatures [72], we can conclude that under Mediterranean climates, there is a higher probability for adults to overwinter.

It is also worth noting that life table experiments are an extreme condition, as in a field reality, insects can select niches more favourable for development: this is a contingent action that increases the survival rate at both low and high temperatures. Winkler et al. [72] showed that limited exposure of the specimens to both high and low temperatures does not significantly affect the life cycle of *D. suzukii*. Conversely, Eben et al. [71] analysed the effect of heat waves on adult populations of *D. suzukii*, observing mortality in up to 80% of older females. This effect was reduced if the individuals were previously acclimated, and this result is partially in line with what we observed at higher temperatures. Our study, in fact, defines the thermal limits in the case where constant conditions are applied since the beginning of the life cycle.

Thermal adaptation could particularly be relevant for the overwintering phase as well, even if it was out of the scope of this study. Since in warmer areas, *D. suzukii* overwinters as an adult, warmer winters endorse adult longevity and survival probability. For the moment, this is just a hypothesis that comes from our results, but further *ad hoc* studies could confirm this aspect as well.

Temperature did not significantly affect the pre-oviposition period. The duration ranged between 1 and 2 days, a very low value compared to the total longevity of adult females. Consequently, in the case of high population densities, where there is a high probability for males and females to mate, females can potentially lay eggs approximately 48 h after emergence. These values are in line with the observations of Ryan et al. [46] carried out on Northwest American populations of *D. suzukii* under different constant-temperature conditions.

Egg production, instead, showed a trend over temperature that is similar to Ryan et al. [46], even if a direct comparison is not possible. Unlike Ryan et al. [46], we considered the average total number of eggs produced by females over temperature instead of the daily number of eggs per day. While conceptually speaking, the two quantities are different, they are strongly correlated, explaining similar trends in the data. The peak of egg production, in our case, was observed around 22.9 °C, while Ryan et al. [46] observed it at 22.87 °C, suggesting that high temperatures are not favourable for the fertility performance of this species. Compared to development and mortality, egg production has a maximum value at lower temperatures than mortality and fertility, explaining the high occurrence of *D. suzukii* in late spring and early autumn in a Mediterranean climate.

Due to its extensive host range, resistance to different climatic conditions, high fertility, and strong dispersing capacity, effective control of *D. suzukii* is an issue. Currently, there are different strategies to control this pest, but they are mainly entrusted with the use of agrochemicals [7]. This action can cause problems, as it is usually carried out on ripening fruits; producers should consider either the life cycle and population dynamics of the pest or the shortage time of the products. To facilitate planning actions, different authors have proposed mathematical models that accurately describe the life cycle of this pest [8,33,35,39,74] and that can be used in the near future as decision support systems.

The results of this study, moreover, can also be helpful in supporting accurate projections of *D. suzukii* distribution in climate change scenarios. For this reason, repeating and comparing life table studies of populations from different areas worldwide is fundamental. It is possible that the phenomena of adaptation to different climate zones may affect life table parameters, leading to different climate change scenarios.

Life table studies, besides providing an in-depth knowledge of the biology of the species and of its thermal response, are fundamental for model parameterisation. The existing literature provided quantitative data to make model applications possible, but the level of accuracy can be increased even more by using the outcomes of this research. We believe that this is a great step forward in providing valuable tools and high-level knowledge either to the scientific community or to users to contain outbreaks of this injurious pest worldwide.

## Figures and Tables

**Table 1:** Life table values of *Drosophila suzukii* preimaginal stages at different constant temperatures. Initial cohorts were composed of 50 eggs laid in a restricted time range. Synthetic data at different temperatures involve the mean development time of each life stage and the corresponding standard deviation (SD) and parameters that identify the shape of the distribution of the data (mode, median, kurtosis, and skewness). The relative survival rate is expressed as the proportion of individuals who survived (reported between parentheses in the last column) over the total number of individuals who developed from the previous stage.

Life Stage	Temperature (±1 °C)	Mean ± SD (Days)	Mode (Days)	Median (Days)	Kurtosis (Days)	Skewness (Days)	Relative Survival Rate
Egg	6	9 ± 1	10	9	−1.21	−0.44	0.26 (13)
	9	5 ± 1	5	5	1.79	1.12	0.38 (19)
	13	3.3 ± 0.8	3	3	−0.46	0.04	0.78 (37)
	18	1.5 ± 0.3	1.6	1.6	0.89	−1.24	0.92 (46)
	20	1.4 ± 0.3	1.7	1.3	0.84	−1.15	0.94 (47)
	24	0.9 ± 0.3	0.6	1	−0.70	0.10	0.98 (49)
	25	0.9 ± 0.3	0.6	0.8	−0.53	0.20	0.96 (48)
	26	0.8 ± 0.2	1	0.8	−0.21	−0.08	0.96 (48)
	27	0.8 ± 0.3	1	1	−0.59	0.03	0.88 (44)
	28	0.9 ± 0.2	1	1	4.19	−2.00	0.78 (39)
	29	1.0 ± 0.2	1	1	0.87	0.92	0.74 (36)
	31	1.2 ± 0.2	1.1	1.2	−0.13	0.63	0.44 (22)
	32	1.6 ± 0.1	1.5	1.6	−0.83	0.71	0.18 (9)
	33	1.75 ± 0.08	--	1.75	--	0	0.06 (3)
Larva 1	6	6.3 ± 0.6	6	6	--	1.73	0.23 (3)
	9	5.6 ± 0.5	6	6	−2.26	−0.39	0.63 (12)
	13	4.5 ± 0.5	5	5	−2.12	−0.14	0.81 (30)
	18	2.0 ± 0.3	2	2	0.71	−0.80	0.85 (40)
	20	1.6 ± 0.2	1.7	1.7	−0.39	0.04	0.82 (40)
	24	1.3 ± 0.2	1.3	1.3	−0.50	−0.02	0.93 (46)
	25	1.2 ± 0.1	1.3	1.3	−0.84	−1.09	0.93 (45)
	26	1.2 ± 0.2	1.3	1.3	−2.01	−0.27	0.95 (46)
	27	1.0 ± 0.05	1	1	41	6.40	0.91 (41)
	28	0.8 ± 0.2	1	1	−2.11	−0.12	0.85 (34)
	29	1.0 ± 0.2	1.1	1	3.45	1.30	0.77 (28)
	31	1.3 ± 0.3	1.2	1.2	0.67	−0.09	0.54 (12)
	32	1.8 ± 0.2	--	1.8	1.50	1.19	0.44 (4)
	33	--	--	--	--	--	0 (0)
Larva 2	6	--	--	--	--	--	0 (0)
	9	5.8 ± 0.4	6	6	1.40	−1.78	0.83 (10)
	13	4.7 ± 0.6	5	5	−0.48	0.23	0.90 (27)
	18	2.8 ± 0.3	3	3	−0.35	−0.43	0.91 (37)
	20	2.5 ± 0.3	2.6	2.5	−0.86	−0.04	0.94 (38)
	24	1.6 ± 0.3	1.7	1.7	0.93	0.96	0.95 (44)
	25	1.4 ± 0.1	1.3	1.3	4.36	2.48	0.94 (43)
	26	1.2 ± 0.2	1.3	1.3	−0.63	0.38	0.89 (42)
	27	1.2 ± 0.2	1.3	1.3	−1.61	−0.69	0.90 (38)
	28	1.1 ± 0.2	1	1	−0.88	−0.19	0.93 (32)
	29	1.4 ± 0.2	1.3	1.3	−0.64	0.18	0.73 (21)
	31	1.7 ± 0.3	1.6	1.7	1.48	0.25	0.58 (7)
	32	--	--	--	--	--	0 (0)
	33	--	--	--	--	--	0 (0)
Larva 3	6	--	--	--	--	--	0 (0)
	9	7.5 ± 0.8	8	8	1.47	−1.50	0.90 (9)
	13	6.6 ± 0.6	7	7	−0.72	0.28	0.92 (25)
	18	2.9 ± 0.2	3	3	−0.88	−0.03	0.97 (36)
	20	2.4 ± 0.2	2.3	2.3	−0.63	−0.14	1 (38)
	24	2.0 ± 0.2	2	2	1.49	0.29	0.97 (43)
	25	1.7 ± 0.2	1.7	1.7	−0.07	0.22	1 (43)
	26	1.5 ± 0.2	1.3	1.3	−0.31	0.88	1 (42)
	27	1.4 ± 0.2	1.3	1.3	−1.47	0.78	0.96 (37)
	28	1.4 ± 0.3	1.3	1.3	−0.44	0.35	0.96 (31)
	29	1.8 ± 0.3	1.7	1.8	−0.07	0.44	0.84 (18)
	31	2.3 ± 0.1	--	2.2	--	0.93	0.43 (3)
	32	--	--	--	--	--	0 (0)
	33	--	--	--	--	--	0 (0)
Pupa	6	--	--	--	--	--	0 (0)
	9	--	--	--	--	--	0 (0)
	13	13.1 ± 0.9	13	13	−0.26	0.55	1 (25)
	18	5.7 ± 0.0	5.7	5.7	−0.58	−0.06	1 (36)
	20	5.3 ± 0.4	5.3	5.3	0.81	0.81	1 (38)
	24	4.6 ± 0.3	4.7	4.7	−0.33	−0.30	1 (43)
	25	4.6 ± 0.2	4.7	4.7	0.39	−0.30	1 (43)
	26	4.4 ± 0.3	4.3	4.3	−0.19	0.30	1 (42)
	27	4.6 ± 0.2	4.7	4.7	0.70	−0.34	1 (37)
	28	4.7 ± 0.3	4.7	4.7	−0.59	−0.07	1 (31)
	29	5.0 ± 0.5	5.5	5.2	−0.78	−0.21	0.94 (17)
	31	--	--	--	--	--	0 (0)
	32	--	--	--	--	--	0 (0)
	33	--	--	--	--	--	0 (0)

**Table 2:** *Drosophila suzukii* adult male and female longevity. Synthetic data at different temperatures involve the mean development time and the corresponding standard deviation (SD), as well as parameters that identify the shape of the distribution of the data (mode, median, kurtosis, and skewness). Unlike the preimaginal stages, the relative survival rate has not been calculated, given that there are no further life stages after the adults, and, accordingly, the calculation does not make sense.

Life Stage	Temperature (±1 °C)	Mean ± SD (Days)	Mode (Days)	Median (Days)	Kurtosis (Days)	Skewness (Days)
Adult males	6	--	--	--	--	--
	9	--	--	--	--	--
	13	22 ± 3	22	22	0.31	0.89
	18	32 ± 5	--	38.7	−1.89	−0.14
	20	40 ± 10	45.3	41.8	−0.02	−0.96
	24	26 ± 7	24.3	25.7	0.10	−0.93
	25	27 ± 2	25.4	27	−0.79	−0.20
	26	24 ± 6	25.4	25.4	5.64	−2.24
	27	25 ± 1	25.4	25.4	−0.23	−0.81
	28	19 ± 8	8.4	17.4	−0.97	0.41
	29	24 ± 1	--	23.4	−0.84	0.56
	31	--	--	--	--	--
	32	--	--	--	--	--
	33	--	--	--	--	--
Adult females	6	--	--	--	--	--
	9	--	--	--	--	--
	13	20 ± 2	20	20	−0.009	−0.17
	18	25.2 ± 0.0	25.3	24.8	3.68	1.17
	20	24 ± 6	25.7	23.3	5.11	1.76
	24	23 ± 3	22.3	22.7	0.10	0.36
	25	23 ± 2	22	23.1	−0.53	0.43
	26	22 ± 2	21.3	21.7	−0.01	0.36
	27	21 ± 1	21.3	21.3	−0.97	−0.15
	28	21 ± 5	19.3	19.3	1.22	0.97
	29	19.9 ± 0.7	20.3	20.3	0.10	−0.96
	31	--	--	--	--	--
	32	--	--	--	--	--
	33	--	--	--	--	--

**Table 3:** Pre-oviposition period and the average total number of eggs produced per female at different temperatures.

Temperature (°C)	Average Pre-Oviposition Period per Female (Days) (±SE)	Average Total Number of Eggs Produced per Female (±SE)
6	--	--
9	--	--
13	1.9 ± 0.3	39 ± 4
18	2.2 ± 0.2	120 ± 10
20	1.5 ± 0.3	160 ± 20
24	1 ± 1	210 ± 10
25	1.6 ± 0.2	190 ± 7
26	1.3 ± 0.2	170 ± 10
27	2.4 ± 0.2	140 ± 3
28	2.2 ± 0.5	110 ± 20
29	2.8 ± 0.4	70 ± 5
31	--	--
32	--	--
33	--	--

**Table 4:** Parameters (±SE) of the best-fitting development rate functions among (2)–(6) for *Drosophila suzukii* preimaginal stages. Temperatures are expressed in °C, while the other parameters are empirical with no biological meaning. The goodness of fit, instead, is expressed by the coefficient of determination $R^{2}$, the number of degrees of freedom NDF, the $\chi^{2}$-value, and the root mean square error RMSE. The best-fit functions are graphically shown in Figure 2, and the complete list of best-fit parameters is reported in Table A1.

Life Stage	Development Rate Function	Best Fit Parameters (±*SE*)	Goodness of Fit Parameters
Egg	Sharpe and De Michele (4)	A=4.78	$R^{2}=0.48$
		B=−113.99	NDF=400
		C=20.44	RMSE=0.40
		D=272.13	$\chi^{2}=0.03$
		E=6.76	
		F=−137.53	
	Lactin-2 (6)	ρ=0.14±0.02	$R^{2}=0.47$
		$T_{M}=33.9\pm 0.6$	NDF=402
		ΔT=6.9±0.7	RMSE=0.40
		λ=−0.3±0.2	$\chi^{2}=1.4\times 10^{-6}$
Larva 1	Logan (2)	ψ=0.08±0.01	$R^{2}=0.78$
		ρ=0.093±0.006	NDF=323
		$T_{M}=32.6\pm 0.2$	RMSE=0.15
		ΔT=1.5±0.2	$\chi^{2}=0.04$
Larva 2	Logan (2)	ψ=0.043±0.008	$R^{2}=0.82$
		ρ=0.116276±0.01	NDF=281
		$T_{M}=32.3\pm 0.2$	RMSE=0.11
		ΔT=2.2±0.4	$\chi^{2}=0.02$
Larva 3	Logan (2)	ψ=0.049±0.008	$R^{2}=0.81$
		ρ=0.104±0.009	NDF=267
		$T_{M}=32.0\pm 0.3$	RMSE=0.09
		ΔT=2.2±0.4	$\chi^{2}=0.14$
	Sharpe and De Michele (4)	A=4.18	$R^{2}=0.81$
		B=−130.61	NDF=267
		C=35.40	RMSE=0.09
		D=713.49	$\chi^{2}=0.18$
		E=7.17	
		F=−148.51	
Pupa	Sharpe and De Michele (4)	A=7.72	$R^{2}=0.84$
		B=−78.76	NDF=252
		C=15.29	RMSE=0.02
		D=8.71	$\chi^{2}=0.003$
		E=10.69	
		F=−106.39	

**Table 5:** Parameters (±SE) of the best-fitting temperature-dependent mortality rate functions between (7) and (8) for *Drosophila suzukii* preimaginal stages. Temperatures are expressed in °C, while the other parameters are empirical with no biological meaning. The goodness of fit, instead, is expressed by the coefficient of determination $R^{2}$, the number of degrees of freedom NDF, the $\chi^{2}$-value, and the root mean square error RMSE. The best-fit functions are graphically reported in Figure 3, and a complete list of the results is reported in Table A2.

Life Stage	Mortality Rate Function	Best Fit Parameters (±*SE*)	Goodness of Fit Parameters
Egg	Bathtub (7)	$a=\left(1.2\pm 0.6\right)\times 10^{-5}$	$R^{2}=0.98$
		$b=\left(-8\pm 5\right)\times 10^{-4}$	NDF=9
		$c=\left(2\pm 1\right)\times 10^{-2}$	RMSE=0.05
		d=−0.3±0.1	$\chi^{2}=3.91\times 10^{-4}$
		e=2.0±0.5	
Larva 1	Bathtub (7)	$a=\left(3.2\pm 0.5\right)\times 10^{-5}$	$R^{2}=0.98$
		$b=\left(-2.4\pm 0.4\right)\times 10^{-3}$	NDF=9
		$c=\left(6\pm 1\right)\times 10^{-2}$	RMSE=0.05
		d=−0.7±0.1	$\chi^{2}=1.19\times 10^{-5}$
		e=3.4±0.4	
Larva 2	Bathtub (7)	$a=\left(5\pm 1\right)\times 10^{-5}$	$R^{2}=0.95$
		$b=\left(3.5\pm 0.9\right)\times 10^{-3}$	NDF=9
		c=0.10±0.02	RMSE=0.10
		d=−1.2±0.3	$\chi^{2}=2.3\times 10^{-6}$
		e=5±1	
Larva 3	Bathtub (7)	$a=\left(5\pm 1\right)\times 10^{-5}$	$R^{2}=0.95$
		$b=\left(3.6\pm 0.8\right)\times 10^{-3}$	NDF=9
		c=0.10±0.02	RMSE=0.11
		d=−1.2±0.3	$\chi^{2}=0.005$
		e=5±1	

**Table 6:** Parameters (±SE) of the best-fitting functions among (2)–(7) for *Drosophila suzukii* adult survival/longevity rates at different temperatures. Temperatures are expressed in °C, while the other parameters are empirical with no biological meaning. The goodness of fit, instead, is expressed by the number of degrees of freedom NDF, the $\chi^{2}$-value, and the root mean square error RMSE. The best-fit functions are plotted in Figure 4, and a complete list of the results is reported in Table A3.

Life Stage	Longevity/Survival Rate Function	Best Fit Parameters (±*SE*)	Goodness of Fit Parameters
Males	Logan (2)	$\psi =\left(1.0\pm 0.7\right)\times 10^{-2}$	NDF=101
		ρ=0.1±0.4	RMSE=0.03
		$T_{M}=30\pm 20$	$\chi^{2}=0.01$
		ΔT=6±4	
	Lactin-1 (5)	ρ=0.10±0.04	NDF=102
		$T_{M}=35\pm 5$	RMSE=0.03
		ΔT=9±3	$\chi^{2}=0.002$
Females	Lactin-1 (5)	ρ=0.10±0.01	NDF=149
		$T_{M}=35\pm 1$	RMSE=0.009
		ΔT=10±1	$\chi^{2}=0.001$

**Table 7:** Best-fit parameters (±SE) of the function (9) describing the *Drosophila suzukii* total egg production at different temperatures. The goodness of fit is expressed by the coefficient of determination $R^{2}$, the number of degrees of freedom NDF, the $\chi^{2}$-value, and the root mean square error RMSE. The best-fit functions are graphically reported in Figure 5.

Best Fit Parameters (±*SE*)	Goodness of Fit Parameters
α=37,000±0	$R^{2}=0.95$
γ=20±40	NDF=6
λ=30±30	RMSE=13.76
τ=4±0	$\chi^{2}=64.41$
δ=22.9±0.2	

## Data Availability

The script and the raw dataset to fully reproduce the results of this study are available at https://github.com/lucaros1190/DSuzukiiLifeTables (accessed on 3 January 2025).

## Supplementary Materials

The following supporting information can be downloaded at: https://www.mdpi.com/article/10.3390/insects16010060/s1, Raw dataset and scripts to fully reproduce the results of this study. Note that the same documents are publicly available at https://github.com/lucaros1190/DSuzukiiLifeTables (accessed on 3 January 2025).

## Appendix A

Complete list of the best-fit parameters estimated for equations (2)–(8) in the case of *D. suzukii*.

**Table A1:** Best-fit parameters (±SE) of the development rate functions (2)–(6) for *Drosophila suzukii* preimaginal stages. Temperatures are expressed in °C, while the other parameters are empirical with no biological meaning. The goodness of fit, instead, is expressed by the coefficient of determination $R^{2}$, the number of degrees of freedom NDF, the $\chi^{2}$-value, and the root mean square error RMSE. The best-fit functions are graphically shown in Figure 2.

Development Rate Function	Life Stage	Best Fit Parameters (±*SE*)	Goodness of Fit Parameters
Logan (2)	Egg	ψ=0.3±0.5	$R^{2}=0.46$
		ρ=0.2±0.8	NDF=402
		$T_{M}=33.2\pm 0.3$	RMSE=0.41
		ΔT=6±3	$\chi^{2}=0.02$
	Larva 1	ψ=0.08±0.01	$R^{2}=0.78$
		ρ=0.093±0.006	NDF=323
		$T_{M}=32.6\pm 0.2$	RMSE=0.15
		ΔT=1.5±0.2	$\chi^{2}=0.04$
	Larva 2	ψ=0.043±0.008	$R^{2}=0.82$
		ρ=0.116276±0.01	NDF=281
		$T_{M}=32.3\pm 0.2$	RMSE=0.11
		ΔT=2.2±0.4	$\chi^{2}=0.02$
	Larva 3	ψ=0.049±0.008	$R^{2}=0.81$
		ρ=0.104±0.009	NDF=267
		$T_{M}=32.0\pm 0.3$	RMSE=0.09
		ΔT=2.2±0.4	$\chi^{2}=0.14$
	Pupa	ψ=0.1±0.3	$R^{2}=0.76$
		ρ=0.1±0.8	NDF=254
		$T_{M}=32.7\pm 0.6$	RMSE=0.02
		ΔT=7±4	$\chi^{2}=2.6\times 10^{-4}$
Brière (3)	Egg	$a=\left(7\pm 1\right)\times 10^{-4}$	$R^{2}=0.46$
		$T_{L}=8\pm 1$	NDF=402
		$T_{M}=33.3\pm 0.5$	RMSE=0.41
		m=1.5±0.2	$\chi^{2}=0.01$
	Larva 1	$a=\left(1.00\pm 0.06\right)\times 10^{-3}$	$R^{2}=0.76$
		$T_{L}=3\pm 2$	NDF=323
		$T_{M}=32.02\pm 0.03$	RMSE=0.16
		m=6±1	$\chi^{2}=0.20$
	Larva 2	$a=\left(1.11\pm 0.05\right)\times 10^{-3}$	$R^{2}=0.80$
		$T_{L}=6\pm 1$	NDF=282
		$T_{M}=31.07\pm 0.04$	RMSE=0.12
		m=6.78±0.00	$\chi^{2}=0.09$
	Larva 3	$a=\left(8.0\pm 0.4\right)\times 10^{-4}$	$R^{2}=0.79$
		$T_{L}=3\pm 2$	NDF=267
		$T_{M}=31.08\pm 0.09$	RMSE=0.09
		m=5±1	$\chi^{2}=0.21$
	Pupa	$a=\left(5\pm 3\right)\times 10^{-5}$	$R^{2}=0.82$
		$T_{L}=7\pm 1$	NDF=254
		$T_{M}=35\pm 2$	RMSE=0.02
		m=1.0±0.2	$\chi^{2}=0.002$
Sharpe and De Michele (4)	Egg	A=4.78	$R^{2}=0.48$
		B=−113.99	NDF=400
		C=20.44	RMSE=0.40
		D=272.13	$\chi^{2}=0.03$
		E=6.76	
		F=−137.53	
	Larva 1	A=4.39	$R^{2}=0.77$
		B=−152.65	NDF=321
		C=44.79	RMSE=0.15
		D=1024.48	$\chi^{2}=0.10$
		E=7.06	
		F=−170.03	
	Larva 2	A=4.49	$R^{2}=0.80$
		B=−132.21	NDF=279
		C=31.37	RMSE=0.11
		D=600.80	$\chi^{2}=0.05$
		E=7.05	
		F=−156.35	
	Larva 3	A=4.18	$R^{2}=0.81$
		B=−130.61	NDF=267
		C=35.40	RMSE=0.09
		D=713.49	$\chi^{2}=0.18$
		E=7.17	
		F=−148.51	
	Pupa	A=7.72	$R^{2}=0.84$
		B=−78.76	NDF=252
		C=15.29	RMSE=0.02
		D=8.71	$\chi^{2}=0.003$
		E=10.69	
		F=−106.39	
Lactin-1 (5)	Egg	ρ=0.165±0.008	$R^{2}=0.46$
		$T_{M}=33.2\pm 0.2$	NDF=403
		ΔT=5.9±0.3	RMSE=0.41
			$\chi^{2}=0.02$
	Larva 1	ρ=0.183±0.006	$R^{2}=0.76$
		$T_{M}=33.9\pm 0.3$	NDF=324
		ΔT=5.4±0.2	RMSE=0.16
			$\chi^{2}=0.11$
	Larva 2	ρ=0.200±0.006	$R^{2}=0.81$
		$T_{M}=33.0\pm 0.2$	NDF=282
		ΔT=5.0±0.1	RMSE=0.11
			$\chi^{2}=0.04$
	Larva 3	ρ=0.185±0.006	$R^{2}=0.80$
		$T_{M}=33.0\pm 0.3$	NDF=268
		ΔT=5.4±0.2	RMSE=0.09
			$\chi^{2}=0.19$
	Pupa	ρ=0.139±0.004	$R^{2}=0.76$
		$T_{M}=32.7\pm 0.3$	NDF=255
		ΔT=7.1±0.2	RMSE=0.02
			$\chi^{2}=2.8\times 10^{-4}$
Lactin-2 (6)	Egg	ρ=0.14±0.02	$R^{2}=0.47$
		$T_{M}=33.9\pm 0.6$	NDF=402
		ΔT=6.9±0.7	RMSE=0.40
		λ=−0.3±0.2	$\chi^{2}=1.4\times 10^{-6}$
	Larva 1	$\rho =\left(2.88\pm 0.06\right)\times 10^{-2}$	$R^{2}=0.77$
		$T_{M}=33.2\pm 0.3$	NDF=323
		ΔT=1.1±0.2	RMSE=0.15
		λ=−1.19±0.03	$\chi^{2}=0.15$
	Larva 2	ρ=0.18±0.02	$R^{2}=0.80$
		$T_{M}=34.0\pm 0.6$	NDF=281
		ΔT=5.6±0.5	RMSE=0.11
		λ=0.00±0.06	$\chi^{2}=0.23$
	Larva 3	$\rho =(2.3125\pm 0.07)\times 10^{-2}$	$R^{2}=0.80$
		$T_{M}=33.0\pm 0.4$	NDF=267
		ΔT=1.4±0.2	RMSE=0.09
		λ=−1.17±0.02	$\chi^{2}=0.22$
	Pupa	ρ=0.03±0.09	$R^{2}=0.82$
		$T_{M}=45\pm 8$	NDF=254
		ΔT=14±2	RMSE=0.02
		λ=−1±2	$\chi^{2}=0.001$

**Table A2:** Best-fit parameters (±SE) of temperature-dependent mortality rate functions (7) and (8) for *Drosophila suzukii* preimaginal stages. Temperatures are expressed in °C, while the other parameters are empirical with no biological meaning. The goodness of fit, instead, is expressed by the coefficient of determination $R^{2}$, the number of degrees of freedom NDF, the $\chi^{2}$-value, and the root mean square error RMSE. The best-fit functions are graphically reported in Figure 3.

Mortality Rate Function	Life Stage	Best Fit Parameters (±*SE*)	Goodness of Fit Parameters
Bathtub (7)	Egg	$a=\left(1.2\pm 0.6\right)\times 10^{-5}$	$R^{2}=0.98$
		$b=\left(-8\pm 5\right)\times 10^{-4}$	NDF=9
		$c=\left(2\pm 1\right)\times 10^{-2}$	RMSE=0.05
		d=−0.3±0.1	$\chi^{2}=3.91\times 10^{-4}$
		e=2.0±0.5	
	Larva 1	$a=\left(3.2\pm 0.5\right)\times 10^{-5}$	$R^{2}=0.98$
		$b=\left(-2.4\pm 0.4\right)\times 10^{-3}$	NDF=9
		$c=\left(6\pm 1\right)\times 10^{-2}$	RMSE=0.05
		d=−0.7±0.1	$\chi^{2}=1.19\times 10^{-5}$
		e=3.4±0.4	
	Larva 2	$a=\left(5\pm 1\right)\times 10^{-5}$	$R^{2}=0.95$
		$b=\left(3.5\pm 0.9\right)\times 10^{-3}$	NDF=9
		c=0.10±0.02	RMSE=0.10
		d=−1.2±0.3	$\chi^{2}=2.3\times 10^{-6}$
		e=5±1	
	Larva 3	$a=\left(5\pm 1\right)\times 10^{-5}$	$R^{2}=0.95$
		$b=\left(3.6\pm 0.8\right)\times 10^{-3}$	NDF=9
		c=0.10±0.02	RMSE=0.11
		d=−1.2±0.3	$\chi^{2}=0.005$
		e=5±1	
Kim and Lee/Son and Lewis (8)	Egg	k=1±0	$R^{2}=0.72$
		$T_{M}=19\pm 1$	NDF=12
		$\rho_{T}=8.6\pm 0.9$	RMSE=0.18
			$\chi^{2}=0.15$
	Larva 1	k=0.9±0.1	$R^{2}=0.62$
		$T_{M}=18\pm 1$	NDF=11
		$\rho_{T}=10\pm 2$	RMSE=0.19
			$\chi^{2}=0.08$
	Larva 2	k=1±0	$R^{2}=0.58$
		$T_{M}=18\pm 1$	NDF=12
		$\rho_{T}=9\pm 1$	RMSE=0.26
			$\chi^{2}=0.24$
	Larva 3	k=1±0	$R^{2}=0.55$
		$T_{M}=18\pm 2$	NDF=12
		$\rho_{T}=9\pm 2$	RMSE=0.29
			$\chi^{2}=0.55$

**Table A3:** Best-fit parameters (±SE) of the functions (2)–(7) describing the *Drosophila suzukii* adult survival/longevity rate over temperature. Temperatures are expressed in °C, while the other parameters are empirical with no biological meaning. The goodness of fit, instead, is expressed by the number of degrees of freedom NDF, the $\chi^{2}$-value, and the root mean square error RMSE. The best-fit functions are plotted in Figure 4.

Longevity/Survival Rate Function	Life Stage	Best Fit Parameters (±*SE*)	Goodness of Fit Parameters
Logan (2)	Males	$\psi =\left(1.0\pm 0.7\right)\times 10^{-2}$	NDF=101
		ρ=0.1±0.4	RMSE=0.03
		$T_{M}=30\pm 20$	$\chi^{2}=0.01$
		ΔT=6±4	
	Females	$\psi =\left(1.0\pm 0.2\right)\times 10^{-2}$	NDF=148
		ρ=0.1±0.1	RMSE=0.01
		$T_{M}=33\pm 2$	$\chi^{2}=0.03$
		ΔT=5±9	
Briere (3)	Males	$a=\left(2\pm 3\right)\times 10^{-6}$	NDF=101
		$T_{L}=0.01\pm 0.04$	RMSE=0.03
		$T_{M}=39\pm 5$	$\chi^{2}=0.008$
		m=0.7±0.2	
	Females	$a=\left(1\pm 8\right)\times 10^{-6}$	NDF=148
		$T_{L}=0.01\pm 0.01$	RMSE=0.009
		$T_{M}=40\pm 10$	$\chi^{2}=0.003$
		m=0.7±0.8	
Sharpe and De Michele (4)	Males	A=5.03	NDF=99
		B=−98.64	RMSE=0.02
		C=11.62	$\chi^{2}=0.007$
		D=7.11	
		E=11.99	
		F=−81.59	
	Females	A=4.94	NDF=146
		B=−111.46	RMSE=0.006
		C=9.46	$\chi^{2}=0.001$
		D=11.23	
		E=12.00	
		F=−92.64	
Lactin-1 (5)	Males	ρ=0.10±0.04	NDF=102
		$T_{M}=35\pm 5$	RMSE=0.03
		ΔT=9±3	$\chi^{2}=0.002$
	Females	ρ=0.10±0.01	NDF=149
		$T_{M}=35\pm 1$	RMSE=0.009
		ΔT=10±1	$\chi^{2}=0.001$
Lactin-2 (6)	Males	$\rho =\left(2.1\pm 0.5\right)\times 10^{-3}$	NDF=103
		$T_{M}=35\pm 0$	RMSE=0.03
		ΔT=2±0	$\chi^{2}=0.04$
		λ=−0.99±0.01	
	Females	$\rho =\left(2.2\pm 0.2\right)\times 10^{-3}$	NDF=150
		$T_{M}=35\pm 0$	RMSE=0.01
		ΔT=2±0	$\chi^{2}=0.02$
		λ=−0.995±0.006	
Bathtub (7)	Males	$a=\left(-2\pm 1\right)\times 10^{-5}$	
		$b=\left(1.4\pm 0.9\right)\times 10^{-3}$	NDF=100
		c=−0.045764±0.03	RMSE=0.02
		d=0.6±0.4	$\chi^{2}=0.001$
		e=−3±2	
	Females	$a=\left(-2\pm 2\right)\times 10^{-6}$	NDF=147
		$b=\left(-2\pm 1\right)\times 10^{-4}$	RMSE=0.006
		$c=\left(6\pm 5\right)\times 10^{-3}$	$\chi^{2}=1.87\times 10^{-5}$
		d=−0.09±0.07	
		e=0.5±0.3	
